# Long-term efficacy and safety of subcutaneous C1-inhibitor in women with hereditary angioedema: subgroup analysis from an open-label extension of a phase 3 trial

**DOI:** 10.1186/s13223-020-0409-3

**Published:** 2020-02-04

**Authors:** Donald S. Levy, Henriette Farkas, Marc A. Riedl, Florence Ida Hsu, Joel P. Brooks, Marco Cicardi, Henrike Feuersenger, Ingo Pragst, Avner Reshef

**Affiliations:** 1 University of California-Irvine, Orange, 705 W La Veta Avenue, Suite 101, Orange, CA 92868 USA; 2 Hungarian Angioedema Reference Center, Third Department of Internal Medicine, Semmelweis University, Budapest, Hungary; 3 School of Medicine, University of California-San Diego, La Jolla, CA USA; 4 Yale University School of Medicine, New Haven, CT USA; 5 IRCCS-ICS Maugeri Milano, University of Milan, Milan, Italy; 6 CSL Behring, Marburg, Germany; 7 Allergy, Immunology and Angioedema Center, Barzilai Medical Center, Ashkelon, Israel

**Keywords:** Hereditary angioedema, Female, Women, HAEGARDA, Estrogen, C1-inhibitor, Childbearing, Pregnancy, Conception

## Abstract

**Background:**

Women with hereditary angioedema due to C1-inhibitor deficiency (HAE-C1INH) experience more frequent and severe angioedema attacks compared with men. Fluctuations in female sex hormones can influence HAE attack frequency and severity. Subcutaneous C1-INH (C1-INH [SC]) is indicated as routine prophylaxis to prevent HAE attacks. In this post hoc subgroup analysis, we evaluated the efficacy and safety of C1-INH (SC) in female subjects with HAE-C1INH enrolled in an open-label extension of the pivotal phase III COMPACT trial.

**Methods:**

In this multicenter, randomized, parallel-arm trial, eligible subjects (age ≥ 6 years with ≥ 4 attacks over 2 consecutive months) received C1-INH (SC) 40 IU/kg or 60 IU/kg twice weekly for 52 to 140 weeks. Analyses of efficacy endpoints were performed for all female subjects and those of childbearing age (age ≥ 15 to ≤ 45 years), including subjects who became pregnant during the evaluation period.

**Results:**

Overall, 91% (69/76) of female subjects were classified as responders (≥ 50% reduction in HAE attacks relative to the pre-study period); 82% experienced < 1 attack/4 weeks. The median number of attacks/month was 0.10, with 96% median reduction in attacks relative to the pre-study period. Results were similar in the subgroup of subjects of childbearing age. Four women who became pregnant during the trial and were exposed to C1-INH (SC) during the first trimester delivered healthy babies with no congenital abnormalities.

**Conclusions:**

C1-INH (SC) prophylaxis was safe and effective in women with HAE-C1INH, including those of childbearing age. Four women exposed to C1-INH (SC) during the first trimester had uneventful pregnancies and delivered healthy babies.

*Trial registration* Clinicaltrials.gov identifier NCT02316353 (Registered December 10, 2014); https://clinicaltrials.gov/ct2/show/NCT02316353.

## Background

Hereditary angioedema (HAE) due to C1-inhibitor (C1-INH) deficiency (HAE-C1INH) is a rare genetic disorder characterized by recurrent, unpredictable, and disabling episodes of edema. Commonly affected sites include the face, limbs, trunk, and submucosal tissues of the gastrointestinal, genitourinary, and upper respiratory tract. The latter includes potentially life-threatening laryngeal attacks [1, 2]. Although HAE-C1INH is an autosomal dominant disorder, published series of symptomatic patients demonstrate a slight female predominance (60%) [2–4].

The HAE-C1INH disease burden is greater in women compared with men; women with HAE-C1INH experience attacks more frequently and their attacks tend to be more severe [2–4]. In a study of 209 patients with HAE-C1INH (female, n = 127; male, n = 82), significantly more women than men (60.7% vs 43.6%; *P *< 0.02) had > 12 attacks per year [2]. In a retrospective study of 193 French patients, 34.4% of attacks reported by women were classified as severe compared with 23.6% of attacks reported by men [4].

It is not clear why women with HAE are more severely affected than men, but estrogen (endogenous and exogenous) likely plays a role. It is known that natural hormonal fluctuations, such as those occurring during puberty, menses, pregnancy, or menopause, affect the course of HAE and the frequency of HAE symptoms [5]. In a survey of women with HAE-C1INH (N = 150), 62% reported disease worsening during puberty and 32% reported worsening during menopause; 35% reported that attacks were triggered by menstruation and 14% by ovulation [5]. Bork et al. also reported that menstruation and ovulation may trigger skin swelling and abdominal pain [2]. In a study by Zotter et al., menstruation, pregnancy, estrogen-containing oral contraceptive use, and ovulation were identified as HAE attack triggers by patients with HAE-C1INH [6].

The effect of pregnancy on disease activity among women with HAE-C1INH varies; some women report improvement or no change while others report worsening [7–9]. In a study of 41 women with HAE-C1INH and 118 pregnancies, almost half (48%) reported worsening of HAE manifestations, while one-third (33%) reported improvement [7]. Another study of 22 women and 35 pregnancies reported that attack rates increased in 83% of pregnancies, with the highest rates occurring during the second and third trimesters [8]. A more recent review of 61 patients and 125 pregnancies found that attacks increased in 59.2% of the pregnancies, improved in 14%, and remained unchanged in 26.4% [9]. Lactation can also increase the frequency of HAE attacks, particularly abdominal attacks [2, 8, 10].

The majority of women with HAE-C1INH report increased disease activity with use of estrogen-containing oral contraceptives or hormone replacement therapy (HRT) [5, 11]. Bork et al. reported that among 32 women with HAE-C1INH, 63% reported new or worsening symptoms after taking oral contraceptives or HRT [11]. In a retrospective study that included 91 women with HAE-C1INH using contraception, symptoms worsened in 80% of women taking combined oral contraceptives (47/59 patients), while 64% of those on progestin-only contraceptives (9/14 patients) reported improvement [5].

The mechanism by which estrogen promotes HAE attacks has not been clearly elucidated. Joseph et al. suggested that interleukin-1 (IL-1), tumor necrosis factor (TNF)-alpha, and estrogen can promote HAE attacks via stimulation of endothelial cells and augmented activation of the prekallikrein-high-molecular-weight kininogen complex (prekallikrein-HMWK) to generate kallikrein and bradykinin [12]. Estrogen has been shown to directly stimulate the local release of heat-shock protein 90 (Hsp90) from endothelial cells, which activates the prekallikrein-HMWK complex to form kallikrein [12]. Kallikrein cleaves from HMWK the nonapeptide bradykinin, a potent inducer of vasodilation and vascular permeability [12]. Activation of vascular endothelial bradykinin B2 receptors is presumed to be the final step in angioedema formation [12, 13]. Therefore, estrogen may also play an important role in regulating bradykinin B2 receptor expression and function [14].

Treatment approaches for HAE-C1INH include on-demand therapy for HAE attacks, short-term prophylaxis, and long-term prophylaxis [15]. The 2017 guideline for managing HAE, issued by the World Allergy Organization (WAO) in collaboration with the European Academy of Allergy and Clinical Immunology (EAACI), recommends that long-term prophylaxis be considered for “patients who face events in life that are associated with increased disease activity [15].” Because HAE tends to be more frequent, severe, and related to hormonal fluctuations in women, many women may benefit from long-term prophylactic therapy, which is intended to lessen the burden of disease, and reduce the frequency and severity of HAE attacks [15].

The 2017 WAO/EAACI guideline recommends plasma-derived (pd) C1-INH as a first-line option for long-term prophylaxis in patients with HAE-C1INH [15]. Subcutaneous C1-INH (C1-INH [SC], HAEGARDA^®^, CSL Behring, Marburg, Germany) has been approved by the US Food and Drug Administration for routine prophylaxis to prevent HAE attacks in adolescents and adults [16]. Indeed, the efficacy and safety of C1-INH (SC) have been demonstrated in the placebo-controlled, phase III COMPACT trial and an open-label extension (OLE) of that trial, in which subjects were treated for up to 2.7 years [17, 18].

Women comprised the majority of subjects in the COMPACT (Clinical Study for Optimal Management of Preventing Angioedema with Low-Volume Subcutaneous C1-Inhibitor Replacement Therapy) trial (67%) and the OLE (60%) [17, 18]. In the COMPACT trial, 71% of subjects (n = 32/45) randomized to receive the FDA-approved dose of 60 IU/kg C1-INH (SC) were women (mean age, 35.0 ± 13.8 years) [18, 19].

An analysis of that subgroup showed a 93% median reduction in attack rates relative to placebo (median, 4.06 attacks/month with placebo vs 0.29 attacks/month with C1-INH [SC]); 12 of 32 (37.5%) female subjects were attack free during prophylaxis [19]. In addition, responder analyses showed 89% (n = 24/27) had ≥ 50% reduction in the time-normalized number of HAE attacks relative to placebo and were classified as responders; 82% (n = 22) had ≥ 70% reduction in attacks and 52% (n = 14) had ≥ 90% reduction in attacks [19].

The purpose of this post hoc analysis was to evaluate the long-term efficacy and safety of C1-INH (SC) in women with HAE-C1INH enrolled in the OLE, with a special focus on women of childbearing age. Women in this age group may become pregnant during prophylactic therapy, underscoring the need for a safe and effective therapy in this population.

## Methods

### COMPACT OLE trial description

**Fig. 1:**
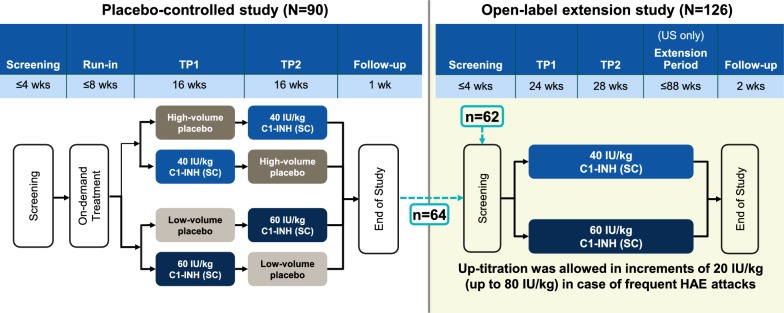
COMPACT OLE trial design. *HAE* hereditary angioedema, *TP* treatment period

The OLE of the COMPACT trial was a multicenter, randomized, parallel-arm trial and included subjects who had completed the COMPACT trial, as well as C1-INH (SC)-naive subjects. Eligible subjects (age ≥ 6 years with ≥ 4 attacks over 2 consecutive months before enrollment) were randomly assigned to receive C1-INH (SC) 40 IU/kg or 60 IU/kg twice weekly for 52 weeks. In the United States, subjects were able to continue treatment for 88 additional weeks (Fig. 1) [18].

The OLE study (NCT02316353) on which the present subgroup analysis is based was done in accordance with the standards of Good Clinical Practice as defined by the International Council for Harmonization of Technical Requirements for Registration of Pharmaceuticals for Human Use, ethical principles that have their origin in the Declaration of Helsinki, and applicable national and local regulations. Study Protocol and amendments were approved by independent ethics committees or institutional review boards at all participating centers prior to study commencement. All patients, or their legal guardians, provided written informed consent.

### Efficacy analyses

The primary objective of the OLE was to assess the long-term safety of C1-INH (SC). Efficacy endpoints were the percentage of subjects with ≥ 50% reduction in attacks relative to the pre-study value (i.e., the number of attacks used to qualify for enrollment into the COMPACT trial or the OLE) and the percentage of subjects with < 1 attack per 4-week period. Exploratory efficacy endpoints included the time-normalized number of HAE attacks and time-normalized rescue medication use [18].

In the OLE, female subjects of childbearing potential were required to use reliable contraception or be sexually abstinent during the trial. Per study protocol, women who became pregnant during treatment were discontinued from the trial. These subjects were included in the efficacy and safety analyses until they discontinued treatment.

Subgroup analyses of efficacy endpoints were performed for all female subjects and those of childbearing age (age ≥ 15 to ≤ 45 years) treated in the OLE, including those who became pregnant during the evaluation period.

Urine samples for pregnancy testing were obtained (1) before randomization and dosing; (2) at weeks 9, 25, 37, and 53 of the OLE; and (3) at weeks 0 (if applicable), 24, 48, 72, and 88 of the additional US extension. Women who became pregnant were followed post-discontinuation until delivery to assess pregnancy outcomes.

## Results

### Baseline demographic characteristics

Of the 126 subjects treated with C1-INH (SC) in the OLE trial, 76 (60.3%) were women (mean age, 40.9 years; mean body mass index [BMI], 28.0 kg/m^2^). Of the 76 female subjects, 42 (55%) were of childbearing age (mean age, 32.5 years; mean BMI, 27.0 kg/m^2^) (Table 1).

**Table 1:** Demographic characteristics of female subjects treated in the OLE of the COMPACT trial

	All female subjects (N = 76)		Females of childbearing age (n = 42)	
	40 IU/kg (n = 40)	60 IU/kg (n = 36)	40 IU/kg (n = 21)	60 IU/kg (n = 21)
Age, mean (SD), y	41.4 (15.2)	40.3 (15.2)	33.1 (9.0)	31.8 (8.3)
Weight, mean (SD), kg	77.6 (23.0)	75.2 (22.6)	77.9 (26.5)	72.1 (21.2)
BMI, mean (SD), kg/m^2^	28.5 (7.9)	27.5 (8.1)	28.0 (9.0)	26.0 (7.8)

*OLE* open-label extension

### Efficacy outcomes in the female study population

Key efficacy outcomes in the female study population and females of childbearing age are presented in Table 2. Overall, 91% of female subjects and 90% of females of childbearing age were classified as responders to treatment with C1-INH (SC), with ≥ 50% reduction in attacks relative to the pre-study period. In the overall population (women and men), 93% of evaluable subjects were responders [18]. In the OLE, 82% of female subjects overall, 81% of female subjects of childbearing age, and 83% of the overall population experienced < 1 attack per 4-week period with C1-INH (SC).

**Table 2:** Secondary efficacy endpoints in female subjects treated long-term with C1-INH (SC)

	All female subjects (n = 76)	Females of childbearing age (n = 42)	Overall study population (N = 126)^18^
Response (≥ 50% reduction in attacks)	91% (69/76)	90% (38/42)	93% (113/122)
< 1 attack/4 weeks	82% (62/76)	81% (34/42)	83% (104/126)

*C1-INH (SC)* subcutaneous C1-inhibitor

During treatment with C1-INH (SC) (40 IU/kg and 60 IU/kg), the median number of HAE attacks per month was 0.10 in female subjects overall, with a 96% median reduction in attacks relative to the pre-study period (Table 3). In female subjects of childbearing age, the median number of attacks per month was 0.16, with a 95% median reduction in attacks relative to the pre-study period.

**Table 3:** Attack frequency, rescue medication use, and attack severity in the female study population with HAE-C1INH

	All female subjects (n = 76)	Female subjects of childbearing age (n = 42)	Overall study population (N = 126)^18^
Number of attacks/month			
Mean (SD)	0.50 (0.86)	0.55 (0.88)	0.45 (0.80)
Median (range)	0.10 (0.0–4.0)	0.16 (0.0–4.0)	0.09 (0.0–4.0)
Rescue medication use/month			
Mean (SD)	0.33 (0.78)	0.30 (0.76)	0.29 (0.70)
Median (range)	0.0 (0.0–4.5)	0.0 (0.0–4.5)	0.0 (0.0–4.5)
Average severity of attacks^a^			
Mean (SD)	1.85 (0.44)	1.89 (0.33)	1.69 (0.56)
Median (range)	2.00 (1.0–3.0)	2.00 (1.0–2.5)	1.64 (1.0–3.0)

*HAE-C1INH* hereditary angioedema due to C1-inhibitor deficiency

^a^1 = mild, 2 = moderate, 3 = severe

Mean (SD) rescue medication use per month was 0.33 (0.78) in the subpopulation of female subjects and 0.30 (0.76) among female subjects of childbearing age, similar to that observed in the overall study population (0.29) (0.76) (Table 3).

### Outcomes in subjects who became pregnant during treatment with C1-INH (SC)

Four women became pregnant within 1 year of starting treatment and were discontinued from the trial, as mandated by the study protocol [18]. In these subjects, C1-INH (SC) exposure after the last menstrual period ranged from 4 weeks (9 doses) to 8 weeks (15 doses); 3 subjects had been treated with the 60 IU/kg dose. Pregnancy was normal in all cases, with no related complications. All 4 women delivered healthy babies (mean weight: 3.1 kg) (Table 4), and no congenital abnormalities were reported during the early postnatal hospital discharge.

**Table 4:** C1-INH (SC) exposure and pregnancy outcomes in subjects with HAE-C1INH who became pregnant during treatment in the OLE

Subject	Age (y)	C1-INH (SC) dose	Total exposure in the OLE	Exposure after last menstruation	Delivery gestation time	Pregnancy outcome
1	19	60 IU/kg	25.3 weeks (42 doses)	8 weeks (15 doses)	Caesarean section at 39 weeks	Healthy baby 3.3 kg
2	27	60 IU/kg	43.1 weeks (87 doses)	5 weeks (10 doses)	Normal forceps at 40 weeks + 5 days	Healthy baby 3.7 kg
3	32	40 IU/kg	35.3 weeks (59 doses)	5 weeks (10 doses)	Caesarean section at 39 weeks + 3 days	Healthy baby 2.9 kg
4	29	60 IU/kg	27.9 weeks (55 doses)	4 weeks (9 doses)	Caesarean section at 36 weeks + 6 days	Healthy baby 2.4 kg

*HAE-C1INH* hereditary angioedema due to C1-inhibitor deficiency, *OLE* open-label extension

**Fig. 2:**
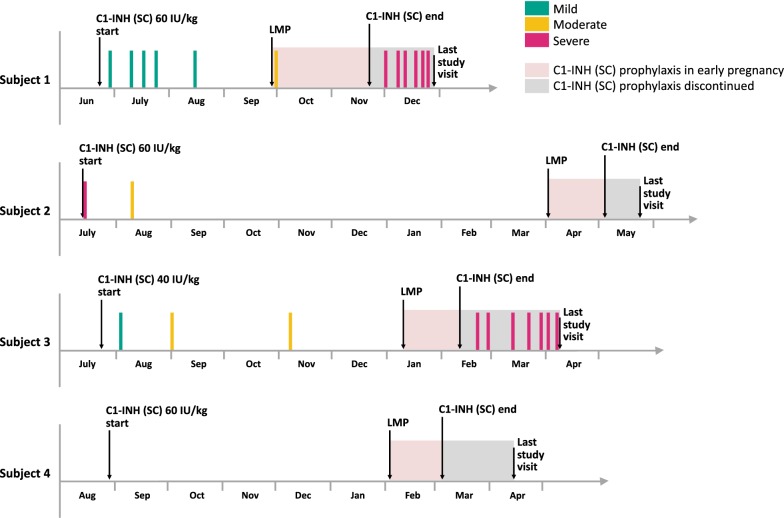
HAE attack patterns in subjects with HAE-C1INH who became pregnant during treatment in the OLE. C1-INH (SC), subcutaneous C1-inhibitor; *HAE* hereditary angioedema, *LMP* last menstrual period, *OLE* open-label extension

During active treatment with C1-INH (SC), all 4 subjects were classified as responders, with ≥ 50% reduction in attacks; all 4 had < 1 attack per 4-week period; 1 subject was attack free. Notably, 3 of the 4 subjects reported no use of rescue medication during prophylaxis with C1-INH (SC), and the remaining subject used rescue medication only once over a period of > 40 weeks of treatment. The 4 women who became pregnant were exposed to C1-INH (SC) for 4 to 8 weeks after their last menstrual period before prophylaxis was discontinued (per study protocol). During this period, 3 subjects had no attacks and 1 experienced a single moderate HAE attack. Immediately after discontinuing C1-INH (SC) prophylaxis, 2 of the 4 subjects reported a sharp increase in the frequency of attacks (Fig. 2). Subject 1 experienced 1 attack between the last menstrual period and treatment discontinuation (8 weeks), but experienced 6 attacks over the 5 weeks between discontinuation of treatment and the last study visit. Subject 3 did not report any attacks between her last menstrual period and treatment discontinuation (5 weeks), but reported 7 attacks in the 8 weeks between treatment discontinuation and the last study visit (subjects did not use any other prophylactic medication during this time period). In both subjects, the rate of attacks between treatment discontinuation and the last study visit was higher than the pre-study rate.

### Safety

The long-term safety profile of C1-INH (SC) in the female study population was consistent with that seen in the overall population (Table 5). Four women discontinued therapy due to adverse events (AEs), including headache (related to study drug), myalgia (related to study drug), arthralgia (not related to study drug), and acute myocardial infarction (not related to study drug). Of the 9 subjects in the trial who had serious AEs, 7 were female and reported 9 of the 12 serious AEs. None of the serious AEs were considered to be related to C1-INH (SC) therapy. The majority of AEs (81%) in female subjects were mild, and 95% of events resolved completely. As in the overall population, injection-site reactions (ISRs) were the most common AE among female subjects. The majority of ISRs were mild, none were severe, and all resolved. No subjects in the OLE had related thromboembolic events; no cases of anaphylaxis were reported; and no subjects had neutralizing anti-C1-INH antibodies at baseline or post-baseline visits [18].

**Table 5:** Adverse event (AE) profile of C1-INH (SC) in female subjects

Adverse events	All female subjects (n = 76)	Females of childbearing age (n = 42)	Overall study population (N = 126)
	No. of events		
Any AE	911	568	1811
AEs leading to discontinuation	4	2	4
Related AEs	505	348	1257
Serious AEs	9	3	12
Related serious AEs	0	0	0
Severity			
Mild	734	473	1572
Moderate	161	87	218
Severe	16	8	21
Outcome			
Recovered/resolved	869	548	1758
Solicited AEs (injection-site reactions			1251
Severity			
Mild	488	339	1234
Moderate	9	5	17
Severe	0	0	0
Outcome			
Recovered/resolved	497	344	1251

*C1-INH (SC)* subcutaneous C1-inhibitor

## Discussion

Women with HAE-C1INH generally have a more severe disease course compared with men, presumably due to the role of estrogens in activating the kallikrein-kinin system [12–14]. Women of childbearing potential may be especially vulnerable, and indeed menstruation and pregnancy have been identified by female patients with HAE as potent triggers of attacks [6]. Various factors unique to women, including menses, ovulation, hormonal contraception, pregnancy, childbirth, breastfeeding, and menopause, lead to fluctuations in estrogen, which can influence HAE attack frequency and severity [5]. Although pregnancy has a variable effect on disease activity, many female patients with HAE experience an increase in attacks during pregnancy [5, 8]. Therefore, many female patients with HAE-C1INH may benefit from prophylactic therapy designed to reduce the frequency and severity of HAE attacks. In this OLE of the pivotal COMPACT trial, C1-INH (SC) was highly effective as long-term prophylaxis in female subjects with HAE-C1INH, including those of childbearing age. Overall, 91% of female subjects had 50% or greater reduction in attacks relative to the pre-study period and 82% had their attack rate reduced to < 1 attack/4 weeks. Among female subjects in the OLE, the median number of attacks per month was 0.10 (~ 1 attack/year), with a 96% median reduction in attacks relative to the pre-study period. C1-INH (SC) was also well tolerated—mild, localized ISRs were the most common AEs.

Prophylactic treatment of HAE in women, especially those of childbearing age, presents some unique challenges. Women of childbearing age may plan a pregnancy or become pregnant unintentionally during HAE prophylactic therapy. Use of an effective therapy that has been proven to be safe during pregnancy may help women with HAE-C1INH in planning a pregnancy and facilitate continuity of HAE management before, during, and after pregnancy.

In current international guidelines, pdC1-INH is the preferred option for long-term prophylaxis in women during pregnancy and lactation [15, 20]. This recommendation is based on several reports documenting the efficacy and safety of pdC1-INH as prophylaxis and acute treatment during pregnancy in women with HAE-C1INH [7–9, 21, 22]. Attenuated androgens are contraindicated in pregnancy and are associated with fetal abnormalities [15, 23]. A review of women exposed to danazol during pregnancy (N = 129 reported pregnancies) showed that of 94 completed pregnancies, 12 resulted in miscarriage, and 23 resulted in the birth of virilized females [23]. Other congenital abnormalities were also reported.

Attenuated androgens are also associated with side effects that can impact conception planning. In a study of patients with HAE-C1INH (N = 118) that included 58 women treated with danazol (2 months to 30 years), the most frequent clinical adverse effects were weight gain, menstrual irregularities (including amenorrhea in 16 of 38 premenopausal women), and virilization [24].

In our study protocol, female subjects of childbearing potential with HAE-C1INH were required to use reliable contraception or be sexually abstinent during the trial. Nevertheless, 4 subjects became pregnant and were exposed to C1-INH (SC) for 4 to 8 weeks after their last menstrual period (i.e., the first trimester of pregnancy). During this period of prophylaxis in early pregnancy, only a single HAE attack was reported. HAE attacks during pregnancy may be especially challenging for women with HAE-C1INH. Abdominal attacks are often associated with nausea, vomiting, and diarrhea [25], which may compound pregnancy-associated gastrointestinal symptoms (“morning sickness”). Abdominal attacks that occur later in pregnancy may be mistaken for labor symptoms. Prevention of HAE attacks with prophylactic therapy may help improve quality of life during pregnancy.

## Conclusions

C1-INH (SC) was demonstrated to be safe and effective as long-term prophylaxis in women with HAE-C1INH. The 4 women who became pregnant during the trial and were exposed to C1-INH (SC) during the first trimester of pregnancy when the risk of teratogenic effects is greatest, delivered healthy babies with no congenital abnormalities. Although C1-INH replacement therapy has been in clinical use for 40 years and its safety and efficacy profile in women with HAE are well established, additional data on the safety of C1-INH (SC) prophylaxis during pregnancy and lactation are needed.

## Data Availability

The datasets used and/or analysed during the current study are available from the corresponding author on reasonable request.

## Abbreviations

C1 INH: C1 inhibitor
COMPACT: Clinical Study for Optimal Management of Preventing Angioedema with Low-Volume Subcutaneous C1-Inhibitor Replacement Therapy
EAACI: European Academy of Allergy and Clinical Immunology
HAE: Hereditary angioedema
HAE-C1INH: Hereditary angioedema due to C1 inhibitor deficiency
HMWK: High-molecular-weight kininogen
HRT: Hormone replacement therapy
Hsp90: Heat shock protein 90
IL: Interleukin
OLE: Open-label extension
pd: Plasma derived
SC: Subcutaneous
TNF: Tumor necrosis factor
TP: Treatment period
WAO: World Allergy Organization
